# Itaconate alleviates thioacetamide-induced acute liver injury by suppressing pyroptosis-associated mitochondrial dysfunction and activating the Nrf2/HO-1 pathway

**DOI:** 10.3389/fphar.2026.1898534

**Published:** 2026-09-14

**Authors:** Lianghong Lv, Wenchang Yang, Qian Yu, Zheng Ma, Song Wang, Yun Lu

**Affiliations:** 1 Department of Gastrointestinal Surgery, the Affiliated Hospital of Qingdao University, Qingdao, China; 2 Tumor Immunology and Cytotherapy of Medical Research Center, Shandong Provincial Key Laboratory of Clinical Research for Pancreatic Diseases, the Affiliated Hospital of Qingdao University, Qingdao University, Qingdao, China

**Keywords:** acute liver injury, GSDMD, itaconate, mitochondrial dysfunction, Nrf2

## Abstract

Acute liver injury is closely associated with inflammatory responses, oxidative stress, hepatocyte death, and mitochondrial dysfunction, whereas effective pharmacological strategies remain limited. This study aimed to investigate the protective effect of 4-octyl itaconate (4-OI) against thioacetamide (TAA)-induced acute liver injury and to further explore the underlying mechanisms. A TAA-induced acute liver injury mouse model, an LPS-stimulated RAW264.7 macrophage inflammatory model, and an LPS/nigericin-stimulated bone marrow-derived macrophage (BMDM) pyroptosis model were established. Liver injury, inflammatory responses, oxidative stress, hepatocyte apoptosis, pyroptosis, and mitochondrial dysfunction were evaluated using histopathological staining, TUNEL staining, biochemical assays, ELISA, qPCR, Western blotting, LDH release assay, immunofluorescence staining, PI staining, MitoSOX staining, and JC-1 assay. ML385 was used to inhibit nuclear factor erythroid 2-related factor 2 (Nrf2) signaling. 4-OI markedly alleviated TAA-induced liver injury, as shown by improved liver histology, reduced hepatocyte apoptosis and macrophage infiltration, decreased serum transaminase levels, and suppressed inflammatory cytokine production. In parallel, 4-OI activated the Nrf2/heme oxygenase-1 (HO-1) pathway, whereas pharmacological inhibition of Nrf2 by ML385 partially reversed its hepatoprotective and anti-inflammatory effects. Increased hepatic GSDMD-N expression after TAA exposure was also reduced by 4-OI. In LPS/nigericin-stimulated BMDMs, 4-OI decreased NOD-like receptor family pyrin domain-containing 3 (NLRP3), cleaved caspase-1, and GSDMD-N expression, accompanied by reduced LDH release, IL-1β secretion, PI-positive cell death, and mitochondrial reactive oxygen species production, as well as restored mitochondrial membrane potential. Moreover, 4-OI reduced mitochondrial GSDMD-N accumulation and attenuated mtDNA-induced pyroptotic activation. These findings indicate that 4-OI alleviates TAA-induced acute liver injury, at least in part, through activation of the Nrf2/HO-1 signaling pathway and suppression of pyroptotic activation and mitochondrial dysfunction-associated inflammatory amplification.

## 1 Introduction

Acute liver injury represents a clinically important pathological condition characterized by rapid hepatocellular damage and abrupt deterioration of liver function. Severe cases may progress to acute liver failure, a life-threatening syndrome occurring in the absence of pre-existing chronic liver disease (Stravitz and Lee, 2019). Multiple etiologies—including drug toxicity, viral infection, ischemia, and autoimmune injury can trigger acute liver injury, yet excessive inflammatory responses and oxidative stress are widely recognized as central events driving hepatic damage progression (Stravitz and Lee, 2019; Allameh et al., 2023). Despite advances in supportive care, effective pharmacological strategies for acute liver injury remain limited. Hence, identification of bioactive molecules capable of attenuating oxidative stress and inflammatory injury holds considerable importance for the prevention and treatment of this condition.

Itaconate, an unsaturated dicarboxylic acid, was first identified as a metabolic product of *Aspergillus terreus* (Calam et al., 1939). Subsequent biochemical studies revealed that itaconate biosynthesis links to citrate metabolism through decarboxylation of cis-aconitate, providing an important basis for understanding its metabolic origin (Bentley and Thiessen, 1957a). Early pharmacological studies reported inhibitory effects of itaconic acid both *in vitro* and *in vivo* (Bo et al., 1952). More recent work further showed that itaconate activates the nuclear factor erythroid 2-related factor 2 (Nrf2) pathway *via* alkylation of Kelch-like ECH-associated protein 1 (KEAP1), and that Nrf2 activation is required for its anti-inflammatory action (Mills et al., 2018). In recent years, itaconate has attracted increasing attention as a bioactive metabolite with anti-inflammatory and cytoprotective potential. Notably, growing evidence suggests its involvement in modulating oxidative stress and inflammatory signaling, positioning it as a promising candidate for intervening in inflammatory tissue injury.

Pyroptosis is a lytic, pro-inflammatory form of programmed cell death mediated by inflammatory caspases and gasdermin family proteins, typically accompanied by membrane pore formation and release of inflammatory mediators such as IL-1β and IL-18 ((Kuriakose and Kanneganti, 2023), (Liu et al., 2024)). Among known upstream signaling platforms, the NOD-like receptor family pyrin domain-containing 3 (NLRP3) inflammasome represents one of the best-characterized pathways driving pyroptosis in inflammatory diseases (Takahashi, 2022). Beyond canonical plasma membrane rupture, recent studies have demonstrated that mitochondrial injury closely associates with pyroptotic signaling and can further amplify inflammasome activation and inflammatory damage (Chen et al., 2022). Based on these observations, pyroptosis-associated mitochondrial dysfunction during inflammatory injury has gradually attracted attention. Thus, suppression of pyroptosis-associated mitochondrial dysfunction may represent an important strategy for alleviating inflammatory liver damage.

Against this background, the present study aimed to investigate the protective effect of itaconate on thioacetamide (TAA)-induced acute liver injury and to further explore the underlying mechanisms. Using a TAA-induced mouse model combined with macrophage-based *in vitro* experiments, the effects of itaconate on liver injury, inflammatory responses, and oxidative stress were evaluated. Special focus was placed on the involvement of Nrf2/heme oxygenase-1 (HO-1) signaling and pyroptosis-associated mitochondrial dysfunction, so as to clarify whether itaconate exerts hepatoprotective effects through regulation of these pathways.

## 2 Materials and methods

### 2.1 Animals and experimental design

Male C57BL/6 mice (7–9 weeks old, weighing 20–26 g) were obtained from the Experimental Animal Center of China Three Gorges University (Hubei, China). All animals were maintained under specific pathogen-free (SPF) conditions with a 12-h light/dark cycle and were provided free access to standard laboratory chow and sterilized water. The experimental animal feed was supplied by Jiangsu Medicine Biotechnology Co., Ltd (China). Mice were acclimated to the laboratory environment for 1 week before experimentation. Mice were randomly assigned into two independent experimental designs. In the first experiment, mice were divided into five groups (n = 6 per group): control group, TAA group (300 mg/kg), TAA + 4-octyl itaconate (50 mg/kg) group, TAA + 4-OI (100 mg/kg) group, and 4-OI alone group (100 mg/kg). This experiment was designed to evaluate the protective effects of 4-OI against TAA-induced acute liver injury. In the second experiment, mice were divided into six groups (n = 6 per group): control group, TAA group (300 mg/kg), TAA + ML385 group, TAA + 4-OI group (100 mg/kg), TAA + 4-OI + ML385 group, and ML385 alone group. This experiment was performed to investigate whether the protective effects of 4-OI were mediated through Nrf2 signaling. The TAA-induced inflammatory injury model has been widely used to investigate mechanisms of acute inflammatory tissue damage (Ezhilarasan, 2023). Previous studies have demonstrated that intraperitoneal injection of TAA reliably induces robust inflammatory responses and oxidative stress, thereby establishing a stable experimental model of inflammatory injury (Zheng et al., 2018). 4-OI was administered by intraperitoneal injection (i.p.) at doses of 50 mg/kg and 100 mg/kg 2 h before TAA challenge. After treatment, mice were sacrificed 24 h later. All animal procedures complied with the NIH Guide for the Care and Use of Laboratory Animals and were approved by the institutional ethics committee. Specifically, all animal experiments received approval from the Animal Ethics Committee of Qingdao University (Approval No. QYFY WZLL 42294 ) and were conducted in accordance with the ARRIVE guidelines and the National Institutes of Health Guide for the Care and Use of Laboratory Animals.

### 2.2 Reagents and antibodies

Itaconate and ML385 were purchased from MedChemExpress (MCE, Shanghai, China); lipopolysaccharide (LPS) was obtained from Sigma-Aldrich (St. Louis, MO, USA). Mouse macrophage colony-stimulating factor (M-CSF) was purchased from PeproTech (Rocky Hill, NJ, USA). ELISA kits for IL-1β, TNF-α, IL-6, MCP, and IL-10 were sourced from Dakewei Biological Engineering Co. (Shenzhen, China). ALT and AST detection kits were obtained from Nanjing Jiancheng Bioengineering Institute (Nanjing, China). The reactive oxygen species (ROS) probe 2′,7′-dichlorodihydrofluorescein diacetate (DCFH-DA), MitoTracker Red CMXRos, lactate dehydrogenase (LDH) cytotoxicity assay kit, mitochondrial membrane potential assay kit with JC-1, DAPI staining solution, malondialdehyde (MDA) assay kit, superoxide dismutase (SOD) assay kit, and glutathione (GSH/GSSG) detection kits were purchased from Beyotime Biotechnology (Shanghai, China). MitoSOX™ Red mitochondrial superoxide indicator and Lipofectamine™ 3,000 Transfection Reagent were obtained from Invitrogen, Thermo Fisher Scientific (Waltham, MA, USA). The TUNEL apoptosis detection kit was purchased from BioLegend (San Diego, CA, USA). The mitochondrial isolation kit was obtained from BioVision (Milpitas, CA, USA). Mitochondrial DNA (mtDNA) was isolated using a Mitochondrial DNA Isolation Kit (ab65321, Abcam, Cambridge, UK).

Primary antibodies against Nrf2, NLRP3, and phospho-p65 (p-p65) were purchased from Cell Signaling Technology (Danvers, MA, USA). Antibodies against HO-1, cleaved caspase-1, and gasdermin D (GSDMD) were obtained from Abcam (Cambridge, UK). The anti-GSDMD-N antibody was purchased from HUABIO (Hangzhou, China), and antibodies against voltage-dependent anion channel 1 (VDAC1) and glyceraldehyde-3-phosphate dehydrogenase (GAPDH) were obtained from Proteintech (Wuhan, China). For immunofluorescence staining, the fluorophore-conjugated secondary antibody used for GSDMD-N detection was goat anti-rabbit IgG H&L (iFluor™ 488, HA1121, HUABIO, Hangzhou, China).

### 2.3 Isolation of bone marrow-derived macrophages

Bone marrow-derived macrophages (BMDMs) were isolated from C57BL/6 mice. Briefly, femurs were collected under sterile conditions, and bone marrow cells were flushed out with phosphate-buffered saline (PBS, Solarbio, Beijing, China). After centrifugation, red blood cell lysis buffer was applied to remove erythrocytes. The remaining cells were cultured in RPMI-1640 medium supplemented with 10% fetal bovine serum (FBS) and 10 ng/mL M-CSF at 37 °C in a humidified 5% CO_2_ incubator. Culture medium was replaced every 48 h, and cells were used for subsequent experiments after 6 days of differentiation.

### 2.4 Cell culture and treatment

RAW264.7 macrophages were maintained in Dulbecco’s Modified Eagle Medium (DMEM) containing 10% FBS at 37 °C in a humidified 5% CO_2_ incubator. For inflammatory stimulation, RAW264.7 cells were pretreated with 4-OI for 1 h and then exposed to LPS (1 μg/mL) for 24 h before further analyses. For pyroptosis induction, BMDMs were primed with LPS (1 μg/mL) for 4 h and then stimulated with nigericin (NG, 10 μM) for 60 min. For 4-OI intervention, cells were pretreated with 4-OI at final concentrations of 50 μM or 100 μM for 1 h before LPS priming, followed by NG stimulation under the same conditions. mtDNA was isolated using a Mitochondrial DNA Isolation Kit (ab65321, Abcam) according to the manufacturer’s instructions. For mtDNA transfection, purified mtDNA and Lipofectamine™ 3,000 Transfection Reagent (Invitrogen, Thermo Fisher Scientific, Waltham, MA, USA) were separately diluted in Opti-MEM™ I Reduced Serum Medium (Gibco, Thermo Fisher Scientific, Waltham, MA, USA), gently mixed, and incubated at room temperature to form transfection complexes. The complexes were then added to BMDMs for the indicated time. Where indicated, cells were pretreated with 4-OI for 1 h before mtDNA transfection.

### 2.5 Histological analysis

For histology, liver tissues were fixed in 4% paraformaldehyde, embedded in paraffin, and sectioned for hematoxylin-eosin (H&E) staining. Histopathological changes were observed and recorded under a light microscope. Liver injury severity was further evaluated using a blinded semi-quantitative Suzuki scoring system according to previously described criteria.

### 2.6 TUNEL staining

TUNEL staining was performed to evaluate hepatocyte apoptosis in liver tissues using a TUNEL apoptosis detection kit (BioLegend, San Diego, CA, USA). Briefly, paraffin-embedded liver sections were deparaffinized, rehydrated, and processed according to the manufacturer’s protocol. After nuclear counterstaining with DAPI, fluorescence images were captured under a fluorescence microscope. TUNEL-positive cells were quantified using ImageJ software and expressed as the percentage of positive cells in randomly selected fields.

### 2.7 Immunofluorescence staining

Paraffin-embedded tissue sections were deparaffinized, rehydrated, and blocked with 5% bovine serum albumin (BSA, Solarbio, Beijing, China). Sections were incubated overnight at 4 °C with anti-F4/80 primary antibody (ab6640, Abcam, Cambridge, UK), followed by incubation with fluorescence-labeled secondary antibodies. Cell nuclei were stained with DAPI, and fluorescence images were captured using a fluorescence microscope and analyzed with ImageJ software. For identification of the cellular source of pyroptotic signals, liver sections were co-stained with GSDMD-N or cleaved caspase-1 and the macrophage marker F4/80.

### 2.8 Immunofluorescence staining for mitochondrial colocalization in BMDMs

BMDMs were cultured to approximately 80% confluence. Following the indicated treatments, cells were incubated with prewarmed MitoTracker Red CMXRos working solution (Beyotime Biotechnology, Shanghai, China) at 37 °C for 15–30 min in the dark. The staining solution was then removed and replaced with prewarmed fresh complete medium, and incubation continued at 37 °C for another 5–10 min. Cells were fixed with 4% paraformaldehyde for 15 min at room temperature, washed with PBS, permeabilized with 0.1% Triton X-100 in PBS for 10–15 min, and blocked with 1% BSA in PBS or PBST for 30–60 min. Thereafter, cells were incubated overnight at 4 °C in the dark with anti-GSDMD-N primary antibody (HUABIO, Hangzhou, China; 1:100). After washing with PBS or PBST, cells were incubated with goat anti-rabbit IgG H&L secondary antibody conjugated with iFluor™ 488 (HA1121, HUABIO, Hangzhou, China; 1:1,000) for 1 h at room temperature in the dark. Nuclei were counterstained with DAPI (Beyotime Biotechnology, Shanghai, China) for 5–10 min. Fluorescence images were captured using a fluorescence microscope and analyzed with ImageJ software.

### 2.9 PI staining

For propidium iodide (PI) staining, cells were collected, washed with PBS, and incubated with PI staining solution at room temperature for 5–10 min. After staining, cells were observed under a fluorescence microscope, and PI-positive cells were quantified using ImageJ software.

### 2.10 ELISA

Levels of inflammatory cytokines, including IL-1β, TNF-α, IL-6, MCP, and IL-10, were measured in mouse serum and in culture supernatants of RAW264.7 cells and BMDMs using commercial ELISA kits according to the manufacturers’ instructions. Briefly, standards or samples were added to ELISA plate wells and incubated at 37 °C, followed by washing steps, incubation with detection antibodies, and addition of substrate solution. The reaction was terminated with stop solution, and absorbance was measured at 450 nm using a microplate reader.

### 2.11 Liver function assay

Serum levels of alanine aminotransferase (ALT) and aspartate aminotransferase (AST) were determined using commercial assay kits following the manufacturers’ protocols.

### 2.12 LDH release assay

LDH release was measured using an LDH cytotoxicity assay kit according to the manufacturer’s protocol, and absorbance was detected at 490 nm with a microplate reader.

### 2.13 ROS measurement

Intracellular ROS levels were detected using DCFH-DA. After the indicated treatments, cells were incubated with 10 μM DCFH-DA at 37 °C for 30 min in the dark. Cells were then washed with PBS to remove excess probe, and the fluorescence intensity of oxidized DCF was measured using a fluorescence microplate reader to assess intracellular ROS production.

### 2.14 Measurement of mitochondrial reactive oxygen species (mtROS)

Following the indicated treatments, cells were incubated with 2.5–5 μM MitoSOX Red at 37 °C for 20 min in the dark, washed with pre-warmed PBS, and fluorescence intensity was measured using a fluorescence microplate reader. The resulting fluorescence intensity was used to evaluate mitochondrial ROS production.

### 2.15 Measurement of mitochondrial membrane potential

Mitochondrial membrane potential was evaluated using a JC-1 assay kit (Beyotime Biotechnology, Shanghai, China) per the manufacturer’s protocol. Following the indicated treatments, cells were incubated with JC-1 working solution at 37 °C for 20 min in the dark, then washed twice with JC-1 staining buffer (1 ×). Red and green fluorescence signals were measured with a fluorescence microplate reader, and the red/green fluorescence intensity ratio served as an indicator of mitochondrial membrane potential.

### 2.16 Oxidative stress assays

Oxidative stress parameters in liver tissues were measured using commercial assay kits. Briefly, liver tissues were homogenized in ice-cold buffer and centrifuged to obtain supernatants. MDA levels, SOD activity, and glutathione-related indices were determined using kits from Beyotime Biotechnology (Shanghai, China). Results were normalized to protein concentration where applicable.

### 2.17 Quantitative real-time PCR

Total RNA was extracted from cells and reverse-transcribed into cDNA according to standard procedures. Quantitative real-time PCR was performed as described previously (Schmittgen and Livak, 2008). Relative mRNA expression was calculated using the 2^−ΔΔCt^ method with GAPDH as the internal control (Livak and Schmittgen, 2001). To evaluate cytosolic mtDNA accumulation, cytoplasmic fractions were isolated after removal of mitochondria using a mitochondrial isolation procedure. DNA was extracted from the cytosolic fraction, and mitochondrial DNA levels were quantified by qPCR using mitochondrial 16 S rRNA as the target gene. HK2 was used as a nuclear DNA marker to normalize mitochondrial DNA signals. (Quiros et al., 2017). Primer sequences were as follows:

GAPDH: forward 5′-AGG​TCG​GTG​TGA​ACG​GAT​TTC-3′, reverse 5′-TGT​AGA​CCA​TGT​AGT​TGA​GGT​CA-3′;

IL-1β: forward 5′-GAT​CCA​CAC​TCT​CCA​GCT​GCA-3′, reverse 5′-CAA​CCA​ACA​AGT​GAT​ATT​CTC​CAT-3′;

IL-6: forward 5′-AGT​CCG​GAG​AGG​AGA​CTT​CA-3′, reverse 5′-ATT​TCC​ACG​ATT​TCC​CAG​AG-3′;

IL-10: forward 5′-AGC​CGG​GAA​GAC​AAT​AAC​TG-3′, reverse 5′-CAT​TTC​CGA​TAA​GGC​TTG​G-3′;

Nrf2: forward 5′-AAC​AAC​GCC​CTA​AAG​CA-3′, reverse 5′-TGG​ATT​CAC​ATA​GGA​GC-3′;

mtDNA (16 S rRNA): forward 5′-ATA​CCC​ATG​GCC​AAC​CTC​CT-3′, reverse 5′-GGG​CCT​TTG​CGT​AGT​TGT​AT-3′;

nDNA (HK2): forward 5′-GCC​AGC​CTC​TCC​TGA​TTT​TAG​TGT-3′, reverse 5′-GGG​AAC​ACA​AAA​GAC​CTC​TTC​TGG-3′

### 2.18 Western blot

Total protein was extracted from tissues or cells using lysis buffer, and protein concentrations were determined *via* BCA assay. For mitochondrial fraction analysis, mitochondrial proteins were extracted from BMDMs using a mitochondrial isolation kit according to the manufacturer’s instructions. Equal amounts of protein were separated by SDS-PAGE and transferred onto PVDF membranes under wet conditions. Membranes were blocked with 5% non-fat milk in TBST for 1 h at room temperature, then incubated with indicated primary antibodies overnight at 4 °C. After washing with TBST, membranes were incubated with corresponding HRP-conjugated secondary antibodies for 1 h at room temperature. Protein bands were visualized using an enhanced chemiluminescence detection system and quantified with ImageJ software, normalized to the respective internal reference protein.

### 2.19 Statistical analysis

Statistical analyses were performed using GraphPad Prism 8 and SPSS 23.0 software. Data are expressed as mean ± standard deviation (SD). Group comparisons were made using Student’s t-test or one-way analysis of variance (ANOVA) followed by Tukey’s *post hoc* test. A *P* value of <0.05 was considered statistically significant.

## 3 Results

### 3.1 Itaconate ameliorates TAA-induced acute liver injury

The *in vivo* effect of 4-OI was first assessed in the TAA-induced acute liver injury model by evaluating liver histopathology, oxidative stress, and inflammatory responses. H&E staining revealed that TAA caused severe hepatic structural disorganization and inflammatory cell infiltration, both of which were markedly ameliorated by 4-OI treatment (Figure 1A). Semi-quantitative evaluation using the Suzuki scoring system further confirmed that TAA induced significant hepatic pathological damage, whereas 4-OI treatment markedly reduced liver injury scores (Figure 1B). Corroborating the histological findings, TAA significantly elevated hepatic MDA levels and reduced GSH and SOD levels, whereas 4-OI effectively reversed these oxidative stress parameters (Figures 1C–E). ELISA analysis further showed that TAA markedly increased the levels of the pro-inflammatory cytokines IL-1β, TNF-α, IL-6, and MCP, and 4-OI treatment significantly lowered these levels (Figures Figureures 1F–I). In contrast, the anti-inflammatory cytokine IL-10, which was decreased in the TAA group, was restored after 4-OI administration (Figure 1J). Collectively, these results demonstrate that 4-OI attenuates TAA-induced acute liver injury, accompanied by suppression of oxidative stress and inflammatory responses.

**FIGURE 1:**
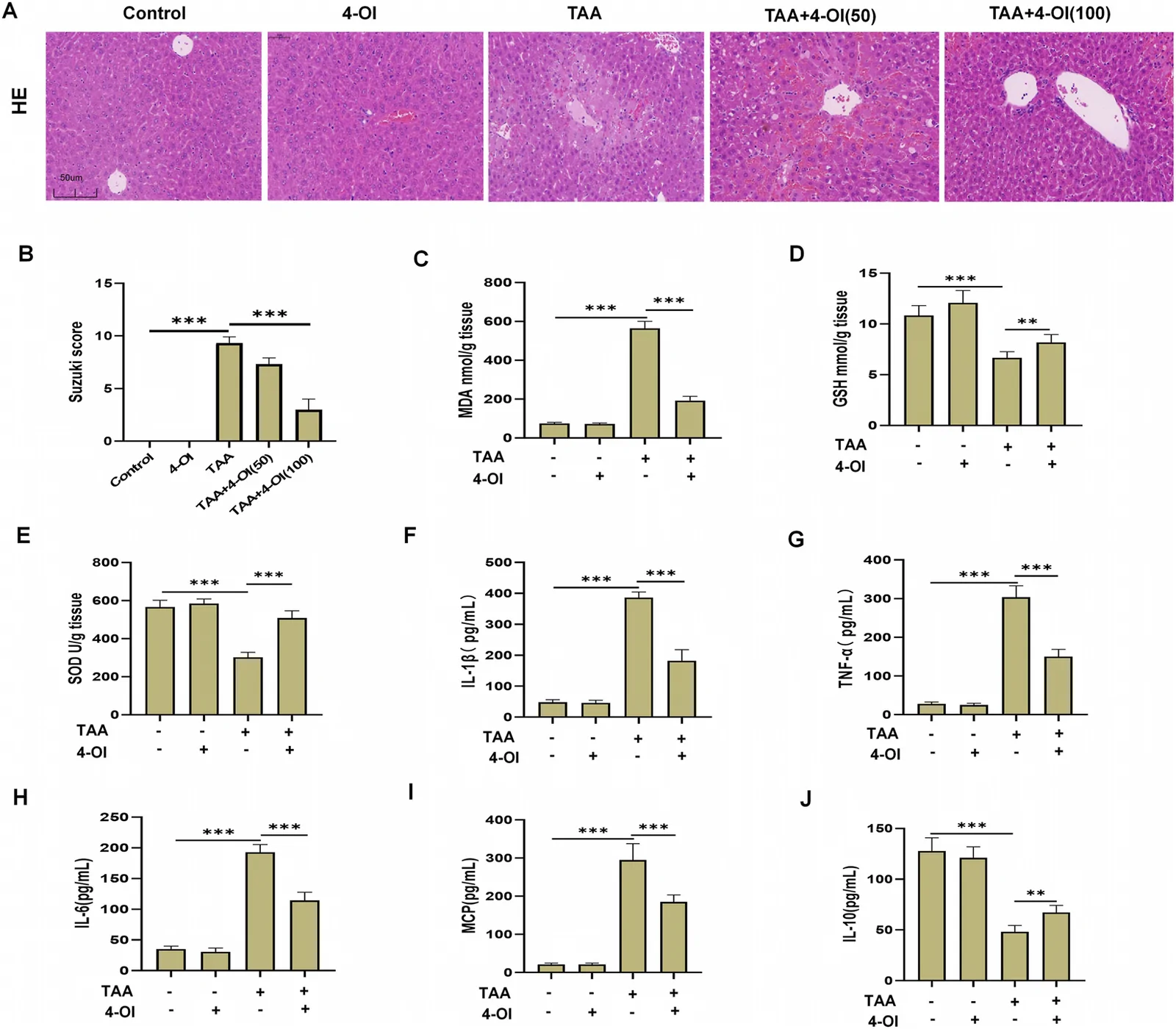
Itaconate ameliorates TAA-induced acute liver injury. **(A)** Representative H&E staining of liver tissues from the indicated groups. **(B)** Semi-quantitative histopathological evaluation of liver injury according to the Suzuki scoring system. **(C–E)** Quantitative analysis of MDA, GSH, and SOD levels in liver tissues. **(F–H)** ELISA analysis of IL-1β, TNF-α, and IL-6 levels in liver tissues. **(I)** Quantification of MCP levels in liver tissues. **(J)** Measurement of IL-10 levels in liver tissues. Data are presented as mean ± SD. Statistical significance is indicated in the figures.

### 3.2 Itaconate reduces hepatocyte apoptosis and macrophage infiltration in liver injury

To further characterize the protective action of 4-OI, hepatocyte apoptosis and macrophage infiltration in liver tissues were examined. TUNEL staining indicated that TAA markedly increased the number of apoptotic hepatocytes, whereas 4-OI treatment significantly reduced the count of TUNEL-positive cells (Figures 2A,B). In parallel, immunofluorescence staining revealed substantial accumulation of F4/80-positive macrophages in the livers of TAA-exposed mice, an effect that was strongly diminished by 4-OI (Figures 2C,D). Thus, 4-OI reduces both hepatocyte apoptosis and macrophage infiltration during TAA-induced liver injury.

**FIGURE 2:**
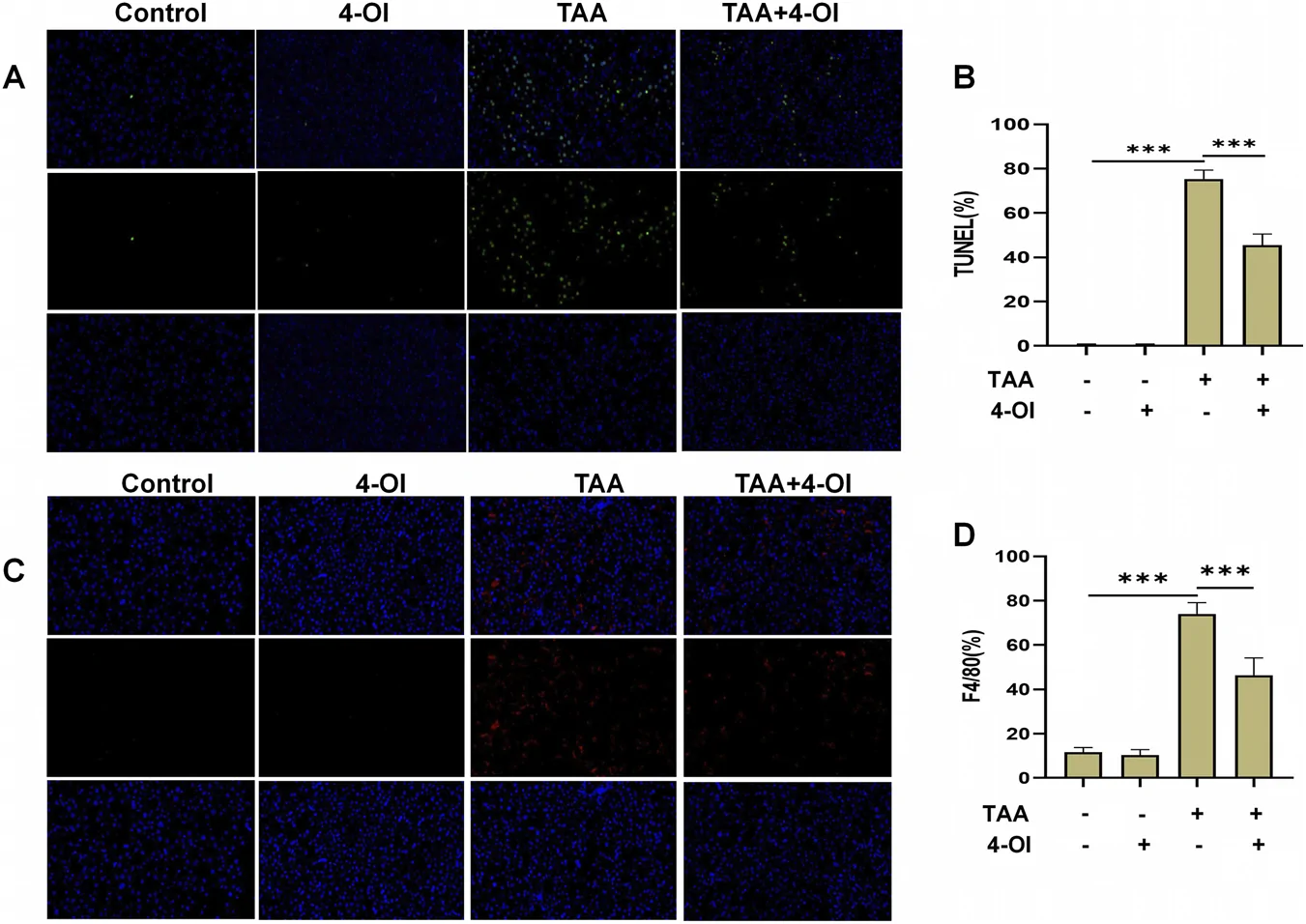
Itaconate reduces hepatocyte apoptosis and macrophage infiltration in TAA-induced liver injury. **(A)** Representative TUNEL staining of liver sections from the indicated groups. **(B)** Quantification of TUNEL-positive cells. **(C)** Immunofluorescence staining of F4/80-positive macrophages in liver tissues. Nuclei were stained with DAPI. **(D)** Quantitative analysis of F4/80-positive cells. Data are presented as mean ± SD. Statistical significance is indicated in the figures.

### 3.3 Itaconate suppresses inflammatory cytokine responses and modulates the nrf2/HO-1 pathway

In LPS-stimulated RAW264.7 macrophages, the cellular response to 4-OI was evaluated by analyzing inflammatory signaling and Nrf2/HO-1 expression. Western blot analysis showed that LPS markedly enhanced p65 phosphorylation, while 4-OI significantly suppressed p-p65 levels (Figures 3A,C). Western blot analysis of nuclear fractions showed that 4-OI treatment increased nuclear Nrf2 accumulation compared with the LPS group (Figure 3B). Simultaneously, LPS enhanced Nrf2 expression under LPS-stimulated conditions, and its downstream target HO-1 was upregulated after 4-OI treatment (Figures 3D,E). Consistent with these protein alterations, qPCR analysis revealed that 4-OI significantly decreased the mRNA levels of IL-1β, IL-6 and IL-10 mRNA expression in LPS-stimulated RAW264.7 cells (Figures 3F–H). ELISA analysis further confirmed that 4-OI reduced IL-1β, IL-6 and IL-10 production (Figures 3I–K). Therefore, 4-OI suppresses LPS-induced cytokine responses and modulates the Nrf2/HO-1 pathway in RAW264.7 macrophages.

**FIGURE 3:**
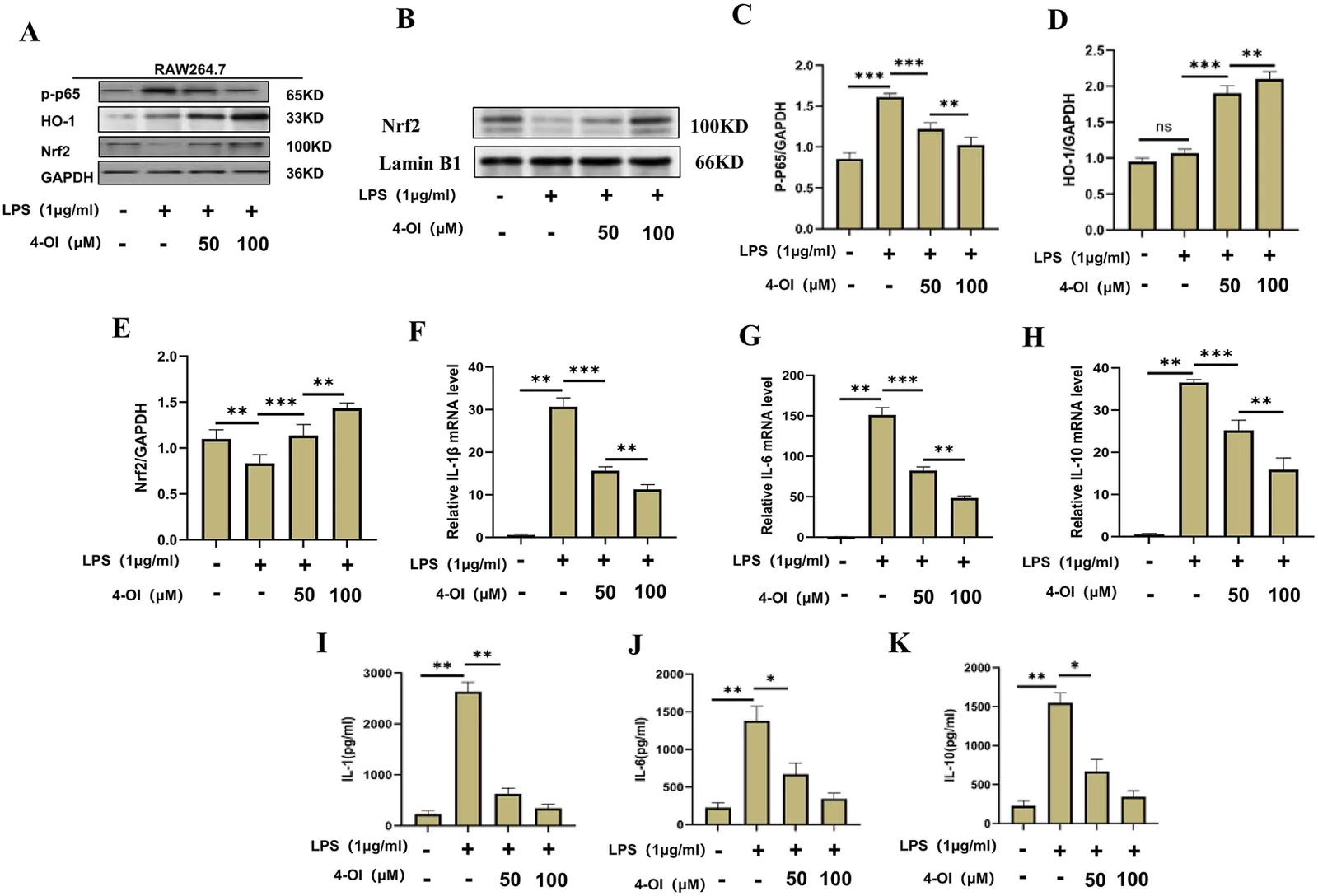
Itaconate suppresses inflammatory signaling and restores Nrf2/HO-1 expression. **(A)** Western blot analysis of p-p65, HO-1, and Nrf2 protein expression. **(B)** Western blot analysis of nuclear Nrf2 levels in RAW264.7 cells. **(C–E)** Densitometric quantification of p-p65/GAPDH, HO-1/GAPDH, and Nrf2/GAPDH ratios. **(F–H)** Relative mRNA levels of IL-1β, IL-6, and IL-10 measured by qPCR. **(I–K)** ELISA analysis of secreted IL-1β, IL-6, and IL-10 levels in cell supernatants. Data are expressed as mean ± SD with statistical significance indicated.

### 3.4 ML385 partially reverses the protective effect of 4-OI in TAA-induced acute liver injury

Given the restoration of Nrf2/HO-1 expression by 4-OI, the contribution of Nrf2 signaling was next examined *in vivo* using the Nrf2 inhibitor ML385. Histological analysis showed that ML385 alone did not induce obvious hepatic injury, whereas ML385 treatment aggravated TAA-induced liver damage and partially abolished the protective effect of 4-OI (Figure 4A) Consistently, blinded semi-quantitative evaluation using the Suzuki scoring system demonstrated that ML385 increased liver injury scores compared with the TAA + 4-OI group, while ML385 alone did not cause obvious pathological changes (Figure 4B). Western blot analysis showed that ML385 treatment significantly reduced hepatic Nrf2 expression compared with the TAA + 4-OI group, confirming the inhibitory effect of ML385 on Nrf2 signaling *in vivo* (Figure 4C). In addition, quantitative analysis of necrotic areas revealed that ML385 increased hepatic necrosis after TAA challenge, whereas 4-OI treatment significantly reduced necrotic lesions (Figure 4D). Consistently, serum ALT and AST levels were significantly elevated after ML385 treatment (Figures 4E,F). ELISA analysis further demonstrated that the levels of IL-1β, TNF-α, and IL-6 were partially restored following Nrf2 inhibition (Figures 4G–I). These findings indicate that ML385 partially reverses the protective effect of 4-OI in TAA-induced acute liver injury.

**FIGURE 4:**
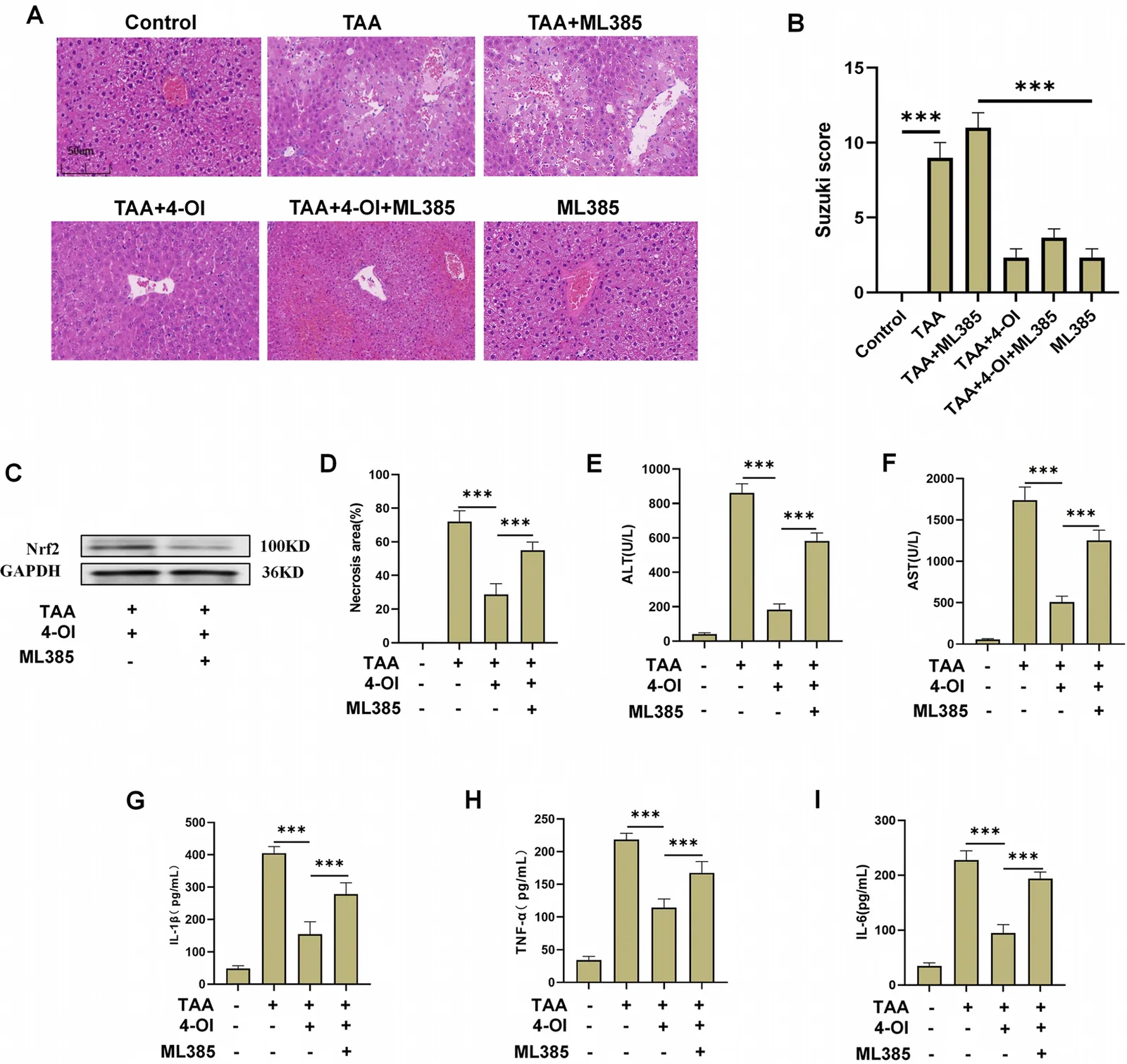
ML385 abolishes the protective effect of itaconate in TAA-induced acute liver injury. **(A)** Representative H&E staining of liver tissues from the indicated groups. **(B)** Semi-quantitative histopathological evaluation of liver injury according to the Suzuki scoring system. **(C)** Western blot analysis of Nrf2 expression in liver tissues from the indicated groups. **(D)** Quantification of necrotic area in liver sections. **(E–F)** Serum ALT and AST levels. **(G–I)** ELISA analysis of inflammatory cytokines, including IL-1β, TNF-α, and IL-6. Data are presented as mean ± SD, and statistical significance is indicated as shown.

### 3.5 Itaconate inhibits pyroptosis in BMDMs

To determine whether pyroptosis participated in the TAA-induced acute liver injury model, GSDMD-N expression in liver tissues from the five experimental groups was further examined by Western blot. Markedly increased GSDMD-N expression was observed in the TAA group, an effect suppressed by 4-OI treatment (Figure 5A), indicating the involvement of GSDMD-mediated pyroptosis in this model. To identify the cellular localization of pyroptotic signals in injured liver tissues, immunofluorescence staining was performed. The results showed that GSDMD-N signals were colocalized with F4/80-positive macrophages in TAA-treated liver tissues, suggesting that macrophages represent an important cellular population associated with pyroptotic activation during TAA-induced liver injury (Figure 5B). Similarly, cleaved caspase-1 signals were also detected in F4/80-positive macrophages and were reduced following 4-OI treatment (Figure 5C). Based on this finding, pyroptosis-related changes were further analyzed in BMDMs. Western blot analysis revealed that LPS + NG markedly upregulated NLRP3, cleaved caspase-1, and GSDMD-N, whereas 4-OI significantly reduced these pyroptosis-associated proteins (Figures 5D,E). Functional assays further demonstrated that pyroptotic stimulation substantially increased IL-1β secretion and LDH release, both of which were markedly decreased by 4-OI (Figures 5F,G). Representative bright-field and PI staining images showed reduced cell injury after 4-OI treatment under pyroptotic conditions (Figure 5H). JC-1 staining indicated that pyroptotic stimulation induced a decrease in mitochondrial membrane potential, which was restored by 4-OI (Figure 5I). In addition, mtROS levels were significantly elevated following pyroptotic stimulation and were markedly reduced by 4-OI (Figure 5J). These results indicate that 4-OI inhibits pyroptosis in BMDMs and that LPS/NG-induced pyroptotic stimulation is accompanied by alterations in mitochondrial status. Based on this observation, whether GSDMD-N is associated with mitochondria under pyroptotic stimulation was next examined.

**FIGURE 5:**
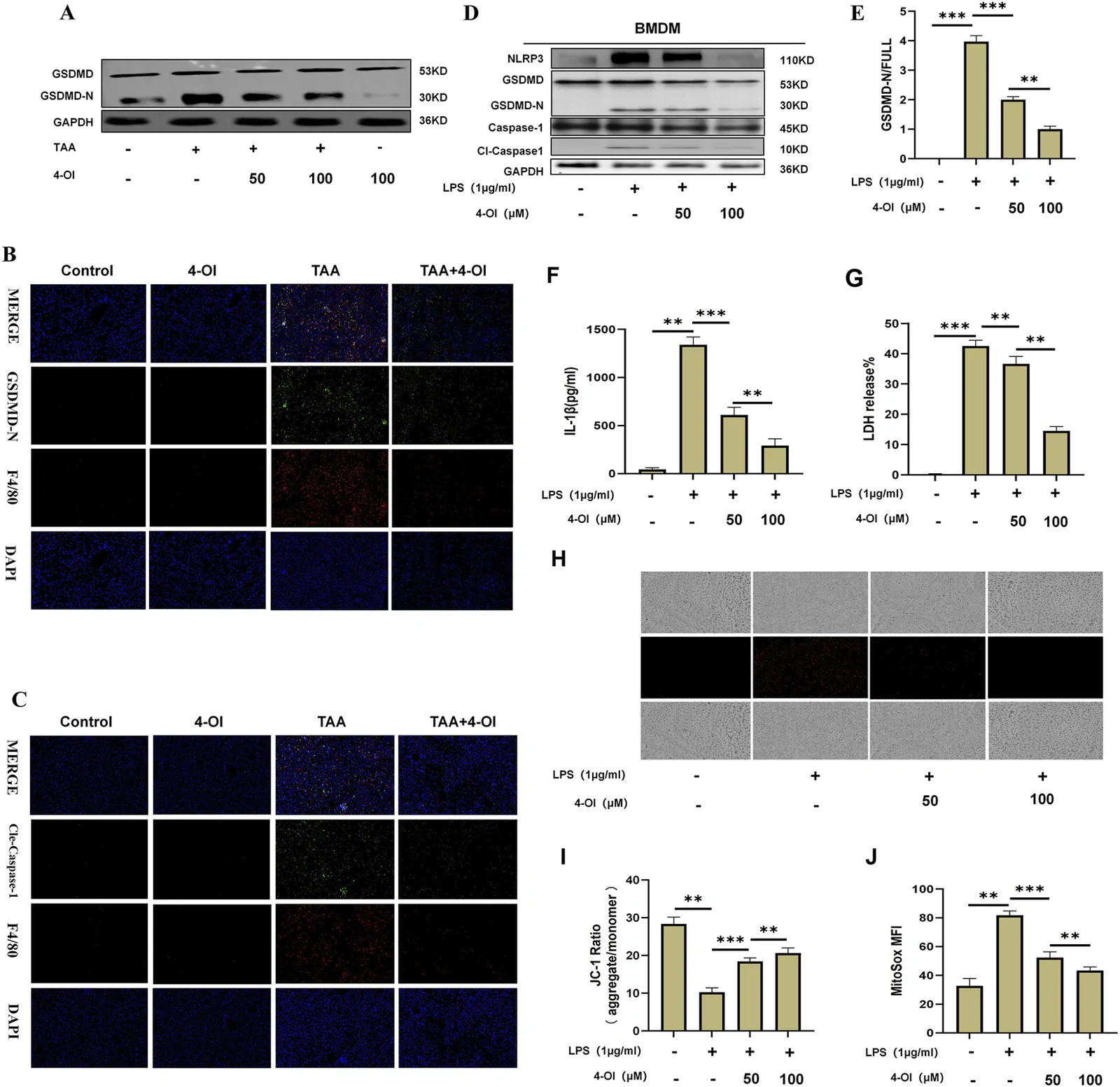
Itaconate inhibits pyroptosis in BMDMs. **(A)** Western blot analysis of GSDMD-N expression in liver tissues from the indicated groups. **(B)** Immunofluorescence staining of GSDMD-N, F4/80, and DAPI showing the colocalization of GSDMD-N with F4/80-positive macrophages in liver tissues. **(C)** Immunofluorescence staining of cleaved caspase-1, F4/80, and DAPI showing the colocalization of cleaved caspase-1 with F4/80-positive macrophages in liver tissues **(D)** Western blot analysis of NLRP3, GSDMD, GSDMD-N, caspase-1, and cleaved caspase-1 protein expression in BMDMs. **(E)** Quantification of the GSDMD-N to full-length GSDMD ratio. **(F)** ELISA analysis of IL-1β secretion. **(G)** LDH release assay to evaluate pyroptotic cell death. Data are presented as mean ± SD. Statistical significance is indicated in figures. **(H)** Representative images of cell morphology and PI staining. **(I)** JC-1 staining to assess mitochondrial membrane potential. **(J)** Measurement of mitochondrial ROS (mtROS) levels.

### 3.6 4-OI attenuates pyroptosis-associated mitochondrial alterations in BMDMs

Given the involvement of GSDMD-mediated pyroptosis in the TAA-induced liver injury model and its confirmation in BMDMs, the association between GSDMD-N and pyroptosis-associated mitochondrial alterations was next investigated. GSDMD executes pyroptosis by forming pores in cellular membranes. Because mitochondria also possess a lipid bilayer membrane structure, it was hypothesized that GSDMD-N might localize to mitochondria and be associated with mitochondrial alterations during pyroptotic stimulation. Immunofluorescence staining revealed increased colocalization of GSDMD-N with mitochondrial signals after stimulation, suggesting an association of GSDMD-N with mitochondria under pyroptotic conditions (Figure 6A). In parallel, Cytosolic mtDNA abundance was significantly increased under inflammatory stimulation. (Figure 6B). Western blot analysis of mitochondrial fractions further demonstrated increased GSDMD-N accumulation under pyroptotic stimulation, whereas 4-OI reduced mitochondrial GSDMD-N expression (Figures 6C,D). These results indicate that 4-OI attenuates pyroptosis-associated mitochondrial alterations in BMDMs.

**FIGURE 6:**
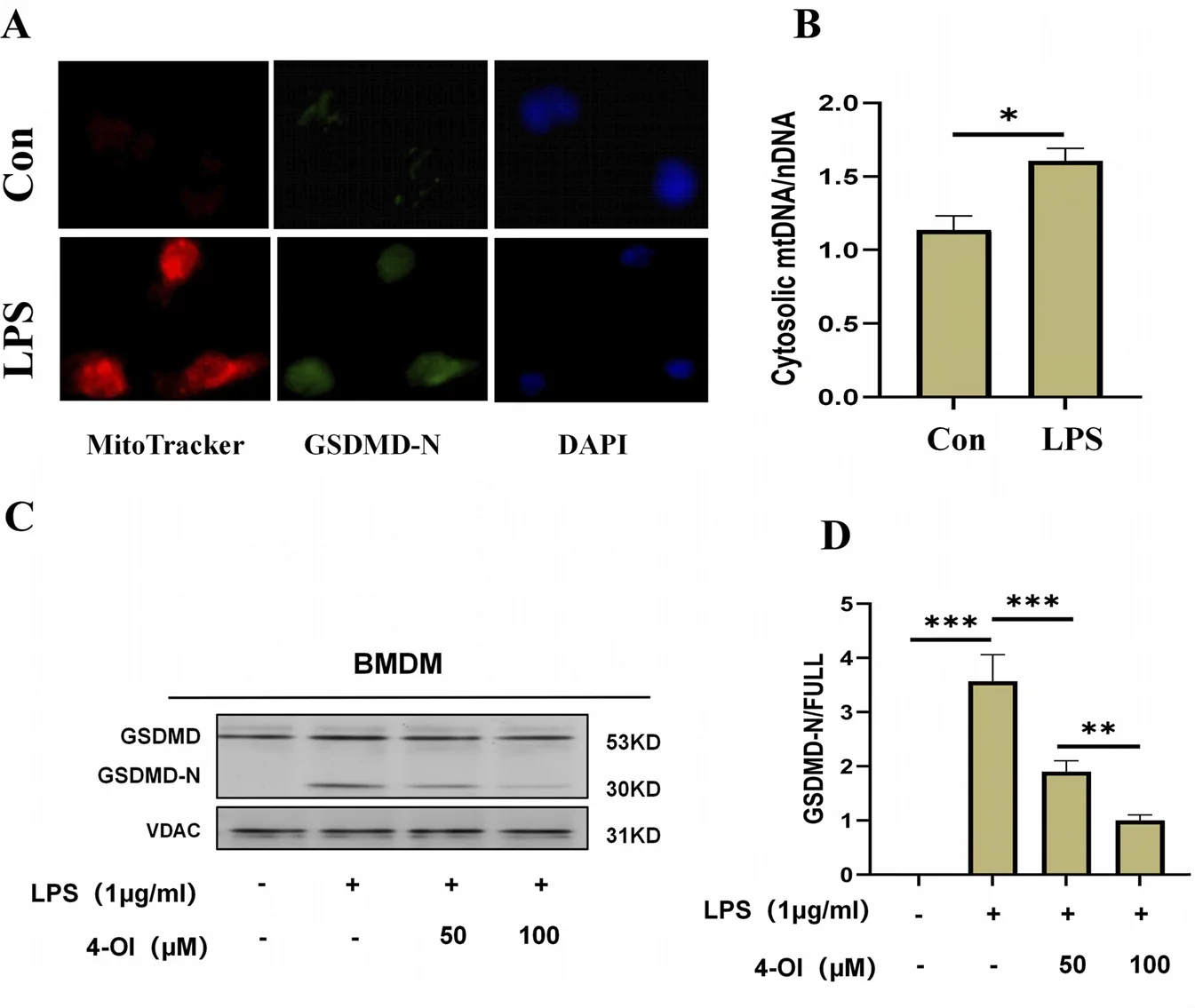
Itaconate attenuates pyroptosis-associated mitochondrial alterations in BMDMs. **(A)** Immunofluorescence staining of MitoTracker, GSDMD-N, and DAPI in BMDMs. **(B)** Quantification of the Cytosolic mtDNA abundance determined by qPCR after mitochondrial removal. **(C)** Western blot analysis of GSDMD and GSDMD-N in mitochondrial fractions of BMDMs. **(D)** Quantification of the GSDMD-N/GSDMD ratio. Data are presented as mean ± SD. Statistical significance is indicated in the figures.

### 3.7 Itaconate attenuates mtDNA-induced pyroptosis in BMDMs

To further explore the relationship between mtDNA-driven inflammatory amplification and pyroptosis, BMDMs were stimulated with mtDNA and analyzed following 4-OI treatment. Western blot analysis showed that mtDNA markedly increased GSDMD-N and cleaved caspase-1 expression, whereas 4-OI significantly reduced both proteins (Figures 7A,B). Functional assays further demonstrated that mtDNA stimulation elevated LDH release and IL-1β secretion, both of which were attenuated after 4-OI treatment (Figures 7C,D).

**FIGURE 7:**
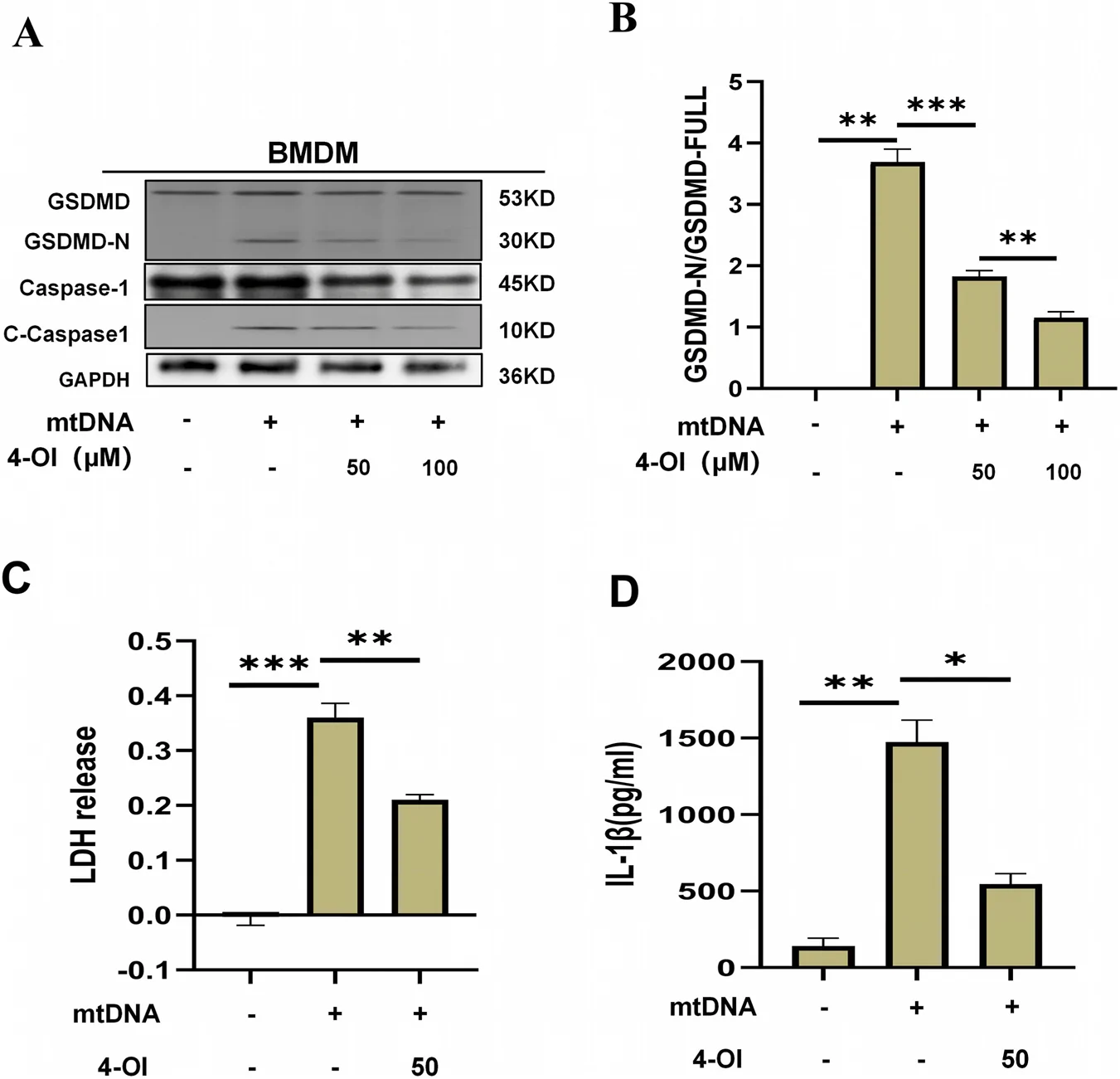
Itaconate attenuates mtDNA-induced pyroptosis in BMDMs. **(A)** Western blot analysis of GSDMD, GSDMD-N, caspase-1, and cleaved caspase-1 protein expression in BMDMs after mtDNA stimulation and 4-OI treatment. **(B)** Quantification of the GSDMD-N to GSDMD ratio. **(C)** LDH release assay. **(D)** ELISA analysis of IL-1β secretion.

## 4 Discussion

Acute liver injury represents a severe pathological condition driven by excessive inflammatory activation, oxidative stress, and hepatocellular death, with severe cases rapidly progressing to liver failure. Current research on acute liver injury has moved beyond general inflammatory damage to focus on the coordinated contributions of programmed cell death, oxidative stress, and organelle dysfunction to disease progression (Stoess et al., 2024; Knorr et al., 2022; Wang et al., 2024). Within this framework, clarifying the interplay among inflammatory amplification, pyroptosis, and mitochondrial damage is essential for understanding pathogenesis and identifying effective therapeutic interventions. In the present study, 4-OI exerted a protective effect against TAA-induced acute liver injury through activation of the Nrf2 pathway and suppression of pyroptosis. GSDMD-N expression was markedly increased in liver tissues after TAA treatment and was reduced by 4-OI, indicating the involvement of GSDMD-mediated pyroptosis in this model. Further *in vitro* experiments showed that 4-OI also alleviated inflammatory responses by reducing pyroptosis-associated mitochondrial alterations.

Itaconate has recently gained increasing attention as an important immunometabolic regulator rather than a simple metabolic intermediate. Historically, itaconic acid was first identified as a metabolic product of *Aspergillus. terreus* (Calam et al., 1939), and subsequent biochemical studies demonstrated that its biosynthesis links to citrate metabolism and the decarboxylation of cis-aconitate (Larsen and Eimhjellen, 1955; Eimhjellen and Larsen, 1955; Bentley and Thiessen, 1957b; Bentley and Thiessen). Later studies further showed that itaconate can be metabolized in mammalian systems and can affect mitochondrial metabolism (Adler et al., 1957; Wang et al., 1961). Early pharmacological work also indicated that itaconic acid possesses inhibitory activity both *in vitro* and *in vivo* (Finkelstein et al., 1947). On this basis, itaconate and its derivatives have been increasingly studied for their roles in redox homeostasis, inflammatory signaling, and macrophage-associated metabolic regulation. Among these derivatives, 4-OIa membrane-permeable derivative with relatively stable biological activity has been widely used to investigate the protective functions of the itaconate pathway. Although beneficial effects of itaconate-related pathways have been reported in multiple inflammatory settings, their role in TAA-induced acute liver injury has remained insufficiently defined. In the present study, 4-OI clearly alleviated TAA-induced hepatic injury *in vivo*, as reflected by improved histopathology, lower biochemical injury markers, and reduced production of inflammatory mediators. These findings were further supported by *in vitro* data from LPS-stimulated macrophages, in which 4-OI suppressed inflammatory activation under stimulated conditions. Thus, the present study extends the protective profile of itaconate derivatives to acute liver injury and supports the concept that modulation of macrophage-associated inflammatory responses constitutes an important component of their therapeutic action.

The Nrf2/HO-1 pathway is a well-recognized cytoprotective axis in inflammatory and oxidative injury. Previous studies have established that oxidative stress closely interacts with inflammasome activation and pyroptotic signaling, and that limiting ROS accumulation can attenuate downstream inflammatory damage (Wang et al., 2024; Wang et al., 2019). Simultaneously, inflammatory cell death and innate immune caspase signaling are also closely associated with ROS-dependent pyroptotic inflammatory responses (Wang et al., 2024; Vasudevan et al., 2023). This background provides a reasonable mechanistic basis for examining whether 4-OI acts through Nrf2/HO-1 in acute liver injury. In the present study, itaconate suppresses inflammatory cytokine responses and modulates the Nrf2/HO-1 pathway. To further verify its contribution, the Nrf2 inhibitor ML385 was introduced. The results showed that ML385 partially reversed the protective effect of 4-OI, as evidenced by aggravated liver pathological injury, elevated ALT and AST, and increased inflammatory cytokine production. These findings suggest that Nrf2/HO-1 signaling represents an important regulatory mechanism contributing to the protective effects of 4-OI. Importantly, ML385 did not itself relieve inflammatory injury in this model; rather, it weakened the beneficial effect of 4-OI. Therefore, a more appropriate interpretation is that 4-OI alleviates acute liver injury, at least in part, through modulation of the Nrf2/HO-1 signaling pathway. Our additional nuclear fraction analysis further demonstrated that 4-OI enhanced nuclear accumulation of Nrf2 under inflammatory conditions, together with increased HO-1 expression, thereby supporting the involvement of Nrf2-associated cytoprotective signaling in limiting downstream inflammatory damage.

Pyroptosis is an inflammatory form of programmed cell death characterized by inflammasome activation, inflammatory caspase cleavage, GSDMD processing, membrane pore formation, and release of inflammatory mediators (Vasudevan et al., 2023; Dai et al., 2023). Excessive pyroptosis can amplify inflammatory responses and contribute to tissue damage in sterile inflammatory diseases (Vasudevan et al., 2023; Rao et al., 2022). In addition, targeting pyroptosis has been considered a potential therapeutic strategy for limiting inflammatory tissue injury (Bandharam et al., 2023). In the present study, pyroptosis was evaluated by examining NLRP3, cleaved caspase-1, and GSDMD-N, together with LDH release, IL-1β secretion, and PI staining. Mitochondrial functional changes associated with pyroptotic stimulation were also assessed by measuring mtROS production and mitochondrial membrane potential. LPS + NG stimulation markedly enhanced all of these pyroptosis-related indicators and induced mitochondrial dysfunction, as shown by increased mtROS levels and decreased mitochondrial membrane potential, whereas 4-OI significantly reduced them. These data demonstrate that 4-OI effectively suppresses pyroptosis in macrophages and alleviates pyroptosis-associated mitochondrial injury. Because pyroptotic cells release inflammatory cytokines and intracellular danger signals that further propagate inflammation, inhibition of pyroptosis likely accounts for an important component of the anti-inflammatory activity of 4-OI in these models (Vasudevan et al., 2023; Broz, 2025).

Compared with classical GSDMD-mediated pyroptosis, the contribution of mitochondrial alterations during pyroptotic injury remains less clearly defined. Classical descriptions of pyroptosis have mainly focused on gasdermin-mediated plasma membrane pore formation and cell lysis (Dai et al., 2023; Broz, 2025), whereas direct participation of mitochondria in pyroptotic injury has not been fully clarified in many inflammatory settings. However, emerging evidence suggests that mitochondrial damage may represent an important component associated with pyroptotic inflammatory responses. Previous studies have shown that mitochondrial ROS and oxidized mitochondrial DNA can promote inflammasome activation and pyroptosis-related inflammatory responses (Wang et al., 2019; Shimada et al., 2012). In addition, mitochondrial ROS production and mtDNA-associated danger signals can amplify inflammasome activation and pyroptosis-related inflammatory injury (Wang et al., 2024; Wang et al., 2019; Shimada et al., 2012). Based on this evidence, we hypothesized that pyroptotic activation may be accompanied by mitochondrial alterations in the present model and that 4-OI may alleviate these changes. To test this possibility, the analysis focused on GSDMD-N localization to mitochondria and mtDNA-associated changes. The results showed that inflammatory stimulation promoted GSDMD-N localization to mitochondria and increased cytosolic mtDNA abundance after mitochondrial removal, indicating mitochondrial damage-associated inflammatory changes. More importantly, 4-OI markedly reduced mitochondrial accumulation of GSDMD-N in mitochondrial fractions, supporting its inhibitory effect on pyroptosis-associated mitochondrial alterations. These findings suggest that 4-OI not only inhibits pyroptotic activation at the cellular level but also attenuates associated mitochondrial abnormalities. Given that mitochondrial damage can sustain a self-amplifying inflammatory loop, preservation of mitochondrial integrity may be especially important for controlling persistent inflammation. Thus, a central implication of this study is that 4-OI may protect against acute liver injury by reducing mitochondrial GSDMD-N accumulation and limiting mtDNA-associated inflammatory amplification.

Several limitations should also be acknowledged. First, although the data consistently support attenuation of pyroptosis-associated mitochondrial alterations by 4-OI, the direct molecular linkage between Nrf2/HO-1 activation and pyroptosis-associated mitochondrial dysfunction remains to be further clarified. Second, the *in vivo* experiments were performed in an acute TAA-induced liver injury model, and whether similar mechanisms operate in chronic liver diseases requires further investigation. Third, only male C57BL/6 mice were used in the present study to minimize biological variability associated with sex-dependent differences in inflammatory responses. However, potential sex-specific effects cannot be excluded, and future studies involving female animals are required to determine whether the protective effects of 4-OI are conserved across sexes. Despite these limitations, the present study systematically demonstrates the protective effect of 4-OI in acute liver injury and provides an integrated explanation involving pyroptotic activation and mitochondrial dysfunction.

## 5 Conclusion

Itaconate alleviates TAA-induced acute liver injury and suppresses inflammatory responses in LPS-stimulated macrophages. These protective effects are closely associated with activation of Nrf2/HO-1 signaling, inhibition of pyroptosis, and, more importantly, attenuation of pyroptosis-associated mitochondrial dysfunction.

## Data Availability

The raw data presented in the study are included in the article/supplementary material, further inquiries can be directed to the corresponding authors.
